# MAPK Signaling Contributes to Primary Follicle Development in Zebrafish (*Danio rerio*)

**DOI:** 10.3390/ani16193078

**Published:** 2026-09-30

**Authors:** Guangjing Zhang, Jing Huang, Xiudan Yuan, Yunpeng Fan, Fangyuan Peng, Wen Fu, Jinhui Liu, Wenbin Liu, Liangyue Peng, Yamei Xiao

**Affiliations:** 1 College of Life Sciences, Hunan Normal University, Changsha 410081, China; zgjing@hnagri.org.cn (G.Z.);; 2 Henan Academy of Fishery Sciences, Zhengzhou 450044, China; 3 Hunan Fisheries Research Institute and Aquatic Products Seed Stock Station, Hunan Academy of Agricultural Sciences, Changsha 410153, China; 4 Agriculture Ministry Key Laboratory of Healthy Freshwater Aquaculture, Key Laboratory of Fish Health and Nutrition of Zhejiang Province, Zhejiang Institute of Freshwater Fisheries (Zhejiang Freshwater Fishery Environmental Monitoring Station), Huzhou 313001, China; 5 Yangtze River Fisheries Research Institute, Chinese Academy of Fishery Sciences, Wuhan 430223, China

**Keywords:** primary follicle, multi-omics analysis, MAPK signaling pathway, zebrafish

## Abstract

Folliculogenesis is essential for fish reproduction, but the role of ERK signaling during early primary follicle development in teleosts remains unclear. Here, we reanalyzed and integrated previously published transcriptomic and DNA methylation datasets from four primary follicle subtypes in zebrafish to identify MAPK-related candidate genes. No new sequencing was performed. Additional qRT-PCR and immunohistochemical analyses examined gene expression and the localization of total ERK and JNK proteins, respectively. Zebrafish were also exposed to the ERK inhibitor SCH772984 from 25 to 60 days post-fertilization to assess changes in ovarian development. The reanalysis identified MAPK-related genes with stage-associated differences in expression and DNA methylation. SCH772984 exposure was associated with reduced ovarian ERK phosphorylation and a lower proportion of advanced follicles. These results support a role for ERK signaling in the progression of early ovarian follicles in zebrafish. These observations derive from a single ERK inhibitor at nominal exposure; complementary assays could strengthen the evidence for target specificity.

## Simple Summary

Early ovarian development depends on the progression of primary follicles (PFs) through distinct developmental stages. However, the signaling pathways involved in these transitions remain unclear in fish. We reanalyzed previously published transcriptomic and DNA methylation datasets from four PF subtypes in zebrafish and conducted additional experiments to examine ovarian development following exposure to the ERK inhibitor SCH772984. Inhibitor exposure was associated with reduced ERK phosphorylation and fewer advanced follicles. These findings suggest that ERK signaling is associated with early follicular development in zebrafish.

## 1. Introduction

In teleosts, folliculogenesis involves oocyte growth, meiosis progression, and coordinated interactions between oocytes and surrounding follicular cells [1,2]. These processes are synergistically modulated by a complex regulatory network composed of endocrine cues, transcriptional regulation, epigenetic modifications, and intracellular signaling cascades [3,4]. Although considerable progress has been made in understanding the hormonal control of final oocyte maturation and ovulation [5], the signaling mechanisms involved in transitions among early primary follicle (PF) subtypes remain less well characterized.

Early PF development is accompanied by changes in nuclear organization, organelle distribution, and follicular-cell structure [6,7]. Our previous study established a discontinuous NaCl-Percoll density-gradient method for isolating four zebrafish PF subtypes, designated PF-i, PF-ii, PF-iii, and PF-iv, according to their cytological characteristics [6,7]. The transition from PF-ii to PF-iii is marked by the appearance of lampbrush chromosomes, dispersal of aggregated mitochondria, and an increase in vesicular structures. These features suggest changes in transcriptional activity and cytoplasmic organization as oocytes grow. Previously published transcriptomic and DNA methylation analyses also revealed substantial molecular differences across this transition, while our proteomic study identified subtype-associated changes involving several signaling pathways, including MAPK [6,7]. Together, these findings provide a basis for investigating the signaling events associated with progression through early PF stages.

The MAPK signaling pathway represents a highly conserved intracellular signaling pathway that modulates multiple biological processes, including cell proliferation, differentiation, apoptosis, metabolism, and stress responses [8]. Among the main downstream components of this pathway, extracellular signal-regulated kinase (ERK) plays a central role in regulating reproductive development [9,10,11]. In mammals, follicle-stimulating hormone (FSH) activates the MAPK/ERK signaling pathway through the FSH receptor, thereby regulating the proliferation of granulosa cells, steroid synthesis, and follicle maturation [12,13]. In avian species, EGFR-dependent activation of the MAPK/ERK cascade facilitates granulosa cell proliferation and inhibits premature follicular atresia [14,15]. These functions are relevant to early follicular growth, which requires coordinated changes in the oocyte and its surrounding somatic cells. In teleosts, however, studies of MAPK signaling have largely focused on meiotic resumption and final oocyte maturation [16,17,18]. Its contribution to earlier PF transitions remains insufficiently understood. The MAPK-related differences detected in our published datasets therefore warrant further examination in developing zebrafish follicles.

In this study, we reanalyzed and integrated previously published transcriptomic and DNA methylation datasets from the four zebrafish PF subtypes to identify MAPK-related candidate genes. Additional qRT-PCR and immunohistochemical experiments examined gene expression and the localization of total ERK and JNK proteins across these subtypes. In a separate experiment, zebrafish were exposed to the ERK inhibitor SCH772984 from 25 to 60 days post-fertilization, followed by assessment of ovarian histology and MAPK-related molecular responses. These analyses were used to examine the involvement of ERK signaling in early follicular progression in zebrafish.

## 2. Materials and Methods

### 2.1. Fish

Wild-type AB strain zebrafish (*Danio rerio*) were maintained at the State Key Laboratory of Developmental Biology of Freshwater Fish, Hunan Normal University, at 26 °C under a 12 h light/12 h dark cycle and fed three times daily. Water pH was maintained at 7.0–7.5 and dissolved oxygen (DO) was ≥6.0 mg/L. Fish age is expressed as days post-fertilization (dpf) throughout this manuscript.

### 2.2. Ethics Statement

Fish work was performed in strict accordance with the recommendations in the Guidelines for the Care and Use of Laboratory Animals of the National Advisory Committee for Laboratory Animal Research in China and was approved by the Animal Care Committee of Hunan Normal University (Permit Number 4237).

### 2.3. Integrative Analysis of Transcriptome and DNA Methylation

Previously published transcriptomic and DNA methylation datasets from four zebrafish primary follicle subtypes—PF-i, PF-ii, PF-iii, and PF-iv—were used for integrative reanalysis (BioProject PRJNA1169999). In the original study, follicles were isolated by discontinuous NaCl–Percoll density-gradient separation and classified using size and cytological characteristics. The reported diameter ranges were 12.6–36.2 μm for PF-i, 34.4–82.8 μm for PF-ii, 60.1–116.8 μm for PF-iii, and 80.9–128.1 μm for PF-iv; representative histological images of each subtype are shown in Figure S1 [7]. Three biological replicates were included per subtype for each assay.

Sequencing was performed on an Illumina platform by Novogene, and the data were processed using HISAT2 v2.0.5 for transcriptomic analysis and Bismark v0.23.1 and cgmaptools v0.1.1 for DNA methylation analysis [7]. The present study used the processed datasets; no new sequencing was performed.

Three adjacent-subtype comparisons were examined: PF-ii versus PF-i, PF-iii versus PF-ii, and PF-iv versus PF-iii, with the earlier subtype serving as the reference. Differentially expressed genes were identified using an adjusted *p* value (padj) ≤ 0.05 and |log_2_(fold change)| ≥ 2.0 and classified as upregulated (RNA_up) or downregulated (RNA_down). Differentially methylated regions were identified using *p* ≤ 0.05 and a methylation difference ≥ 0.20 [7], and genes associated with hypomethylation in CG and CHH contexts were designated CG_down and CHH_down, respectively.

### 2.4. SCH772984 Exposure and Tissue Collection

Zebrafish were assigned to three groups: DMSO vehicle control, 1 μM SCH772984, and 5 μM SCH772984. Healthy and morphologically uniform zebrafish larvae at 25 dpf were selected and randomly allocated to each experimental tank, with approximately 10 larvae per tank. Exposure began at 25 days post-fertilization (dpf) and continued for 35 days, with sampling at 60 dpf.

SCH772984 (selleck, S7101), an ERK1/2 inhibitor characterized in other experimental systems [19,20], was prepared as a 10 mM stock in DMSO. A salt solution containing 0.1 g CaCl_2_, 0.05 g KCl, and 3.5 g NaCl per liter of distilled water was used to dilute the stock during preparation of the exposure solutions. Final nominal SCH772984 concentrations were 1 and 5 μM, and DMSO was adjusted to 0.1% in all groups. To maintain stable drug concentrations throughout the experiment, exposure solutions were completely renewed weekly, and the inhibitor was replenished at each water change according to the nominal concentrations. Preliminary toxicity assays demonstrated that 10 μM SCH772984 exposure caused 100% mortality; therefore, 1 μM and 5 μM were selected as subtoxic concentrations for the formal experiment. Mortality was monitored daily throughout the 35-day exposure period, and no mortality occurred in any experimental group.

At 60 dpf, three fish were sampled per tank, giving 10 fish per group and 30 fish across the three groups. For molecular analyses, ovaries from the three fish within each tank were pooled to form one biological sample. Each group therefore comprised three tank-derived samples (*n* = 3), with the tank serving as the experimental unit. During the sampling process, the body length and weight of the specimens were measured. Quantitative data are presented as mean ± SD.

### 2.5. Quantitative Real-Time PCR (qRT-PCR) Analysis

Total RNA was extracted from PF-i–PF-iv follicles isolated as previously described and from ovarian tissue of DMSO vehicle-control and SCH772984-treated fish using an RNA extraction kit (Magen, Guangzhou, China, R4012). For the exposure experiment, three pooled ovarian samples were prepared per treatment group. Ovaries from three fish within a single tank were pooled to form one sample, so each pooled sample corresponds to one independent tank. RNA was extracted separately from each pooled sample.

Total RNA was digested with DNase I to eliminate genomic DNA contamination. RNA integrity was assessed by agarose gel electrophoresis, and RNA concentration was quantified using a NanoDrop spectrophotometer (Thermo Fisher Scientific, Wilmington, DE, USA). A total of 1 μg of purified total RNA was used as input for each reverse transcription reaction, and cDNA was synthesized using a reverse transcription kit (Takara Bio, Dalian, China).

Primers were designed using the NCBI primer-design tool, screened for potential dimers using Oligo v7, and synthesized by Qingke Bio (Beijing, China) (Table S1). Amplicon lengths and amplification efficiencies (98%) for each primer pair are summarized in Table S1. PCR products were subjected to sequencing. qRT-PCR was performed on a QuantStudio 5 system using 2× ChamQ Universal SYBR qPCR Master Mix (Vazyme, Nanjing, China). Each 20 μL reaction contained 10 μL of master mix, 2.2 μL of cDNA, 0.4 μL each of forward and reverse primers, and 7 μL of double-distilled water. Cycling conditions were 95 °C for 30 s, followed by 40 cycles of 95 °C for 10 s and 60 °C for 30 s. Relative expression was calculated using the 2^−ΔΔCt^ method with β-actin as the sole reference gene.

Statistical analyses were performed using SPSS 20.0 (IBM Corp., Armonk, NY, USA). Molecular measurements among the four PF subtypes were analyzed using one-way repeated-measures ANOVA, with the independent donor pool as the matching unit (*n* = 3). Bonferroni correction was applied to the three adjacent-subtype comparisons: PF-ii versus PF-i, PF-iii versus PF-ii, and PF-iv versus PF-iii.

For the SCH772984 experiment, qRT-PCR outcomes were analyzed using one-way ANOVA, with the tank as the experimental unit (*n* = 3 per group). Each inhibitor group was compared with the vehicle control, with Bonferroni correction for the two comparisons. Quantitative data are presented as mean ± SD.

### 2.6. Histological and Immunohistochemistry

Ovarian tissues collected for histological examination were fixed in Bouin’s solution, dehydrated, and embedded in paraffin. Sections (5–6 μm thick) were cut using a Leica RM2015 microtome (Leica Biosystems, Wetzlar, Germany) and stained with hematoxylin and eosin [21]. Images were captured using a light microscope (Leica Biosystems, Wetzlar, Germany).

Preparation of sections required for immunohistochemistry (IHC). Ovary tissues were fixed in 4% paraformaldehyde dehydrated, embedded in paraffin, sectioned, and deparaffinized with xylene. Antigen retrieval was performed by microwave heating in citrate antigen retrieval solution (Sangon Biotech, Shanghai, China, E673000-0100). Sections were incubated with 3% H_2_O_2_ for 30 min at room temperature in the dark, washed three times with Tris-buffered saline containing Tween-20 (TBST), and blocked with 10% BSA for 30 min at room temperature. Sections were incubated at 4 °C with anti-ERK1/2 (Abmart, Shanghai, China, T40071; 1:200) or anti-JNK1/2/3 (HUABIO, Hangzhou, China, ET1601-28; 1:200), followed by incubation with HRP-conjugated goat anti-rabbit IgG H&L (Abcam, Shanghai, China, ab205718; 1:500) for 1 h at room temperature. After three TBST washes, staining was developed using diaminobenzidine (DAB) and stopped by rinsing with water. Images were captured by light microscopy to examine total ERK and JNK localization. The average optical density (AOD) was quantified using ImageJ software (version 1.54t). Statistical analysis was performed with one-way ANOVA. For this analysis, 3 fish per group were used; one non-consecutive ovarian section was randomly selected from each fish for quantification.

### 2.7. Follicle Quantification and Quantification

Follicle diameters were measured in ovarian sections using ImageJ. Follicles were classified as PF-i, PF-ii, PF-iii, PF-iv, or secondary follicles (SFs) according to size and histological characteristics described previously. The overlapping PF diameter ranges were used alongside cytological features rather than as exclusive classification thresholds. Section-based diameters were not treated as equivalent to measurements of intact isolated follicles.

Three fish were sampled from each of three tanks per treatment group. For each ovary, 3–5 nonconsecutive sections were examined, with 5–10 microscopic fields assessed per section. Counts were combined within each fish to calculate follicle-category proportions, which were then averaged by treatment group. Sections and fields were treated as subsamples, and assessment was performed without blinding. Group means were presented descriptively because the retained percentage values could not be linked to individual fish or tanks.

### 2.8. Western Blot

Pooled ovarian samples were prepared as described in Section 2.4. Proteins were extracted separately from each sample using cell lysis buffer, separated by sodium dodecyl sulfate–polyacrylamide gel electrophoresis, and transferred to nitrocellulose membranes. Membranes were blocked in a blocking solution for approximately 1.5 h, followed by incubation of primary antibodies overnight on a shaker at 4 °C. The primary antibodies were purchased from Huabio and diluted as follows: anti-ERK1/2 (1:1000), anti-p-ERK1/2 (1:2000), anti-JNK1/2/3 (1:1000), anti-p-JNK1/2/3 (1:2000), anti-β-actin (1:1000) and anti-α-tubulin (1:1000). After washing the nitrocellulose membranes, anti-rabbit (Huabio) was diluted at 1:1000 and incubated as appropriate for the primary antibody host species, and chemiluminescence was used for signal detection. Band intensities were quantified using ImageJ. Phosphorylated proteins, their corresponding total proteins, and loading controls were assessed using the same biological samples. The p-ERK/ERK and p-JNK/JNK ratios were calculated for each sample. Statistical analysis of Western blot results from the SCH772984 experiment was performed identically to the qRT-PCR analysis described in Section 2.5.

## 3. Result

### 3.1. Integrative Reanalysis of Transcriptomic and DNA Methylation Profiles Across PF Subtypes

**Figure 1:**
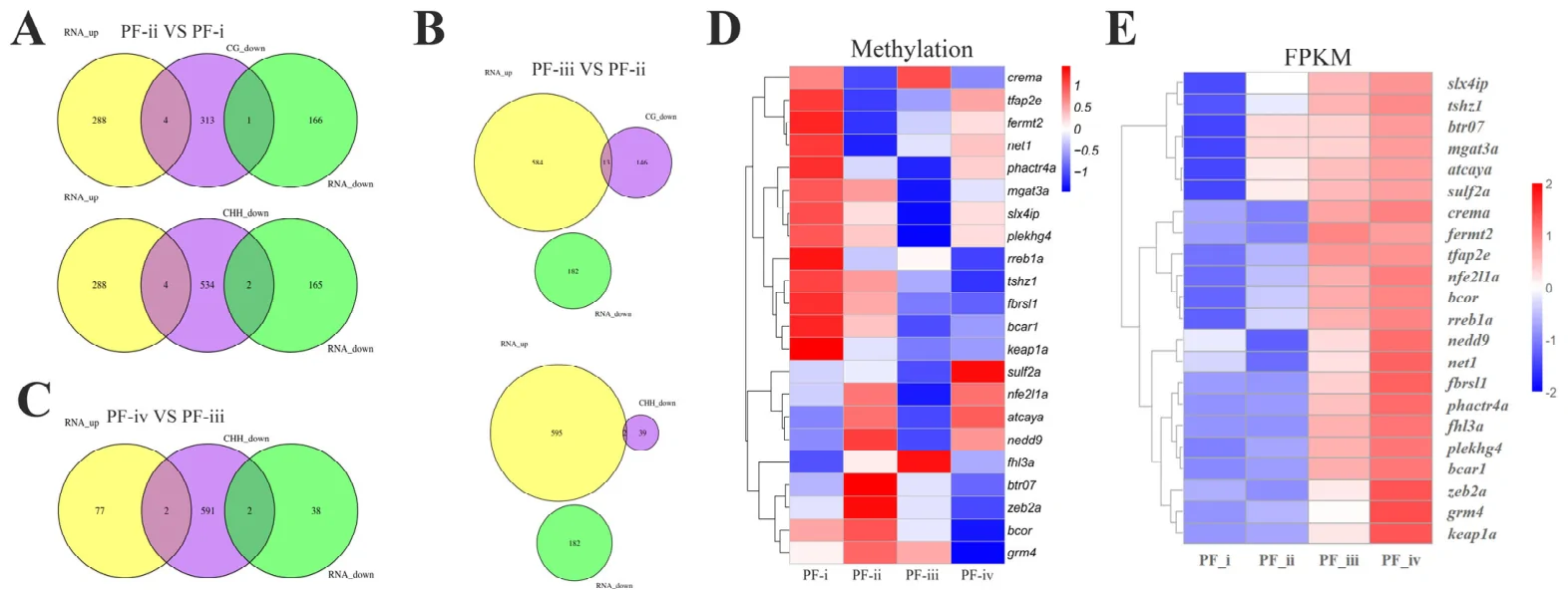
Integrated transcriptomic and DNA methylation analysis show transcriptional and methylation changes during zebrafish primary follicle transition. (**A**–**C**) Venn diagrams showing the overlap between upregulated genes and DNA methylation-downregulated genes during the transitions from PF-i to PF-ii (**A**), PF-ii to PF-iii (**B**), and PF-iii to PF-iv (**C**). (**D**) Heatmap showing the DNA methylation patterns of candidate genes exhibiting stage-specific methylation changes during primary follicle development. (**E**) Heatmap displaying the expression profiles (FPKM values) of corresponding candidate genes across different developmental stages.

We reanalyzed previously published transcriptomic and DNA methylation datasets from four zebrafish primary follicle subtypes [7]. Venn diagrams comparing differentially expressed genes (DEGs) and differentially methylated genes (DMGs) across consecutive developmental stages (PF-ii vs. PF-i, PF-iii vs. PF-ii, PF-iv vs. PF-iii) identified a set of genes exhibiting simultaneous alterations in transcriptional abundance and DNA methylation status (Figure 1A–C). Functional annotation grouped these overlapping genes into multiple core biological functional categories, including transcriptional regulatory factor (TRF) (*bcor*, *hivep2a*, *tfap2e*, *crema*, *zeb2a*, and *nfe2l1a*), protein modification (*btr07*, *mgat3a*, *atcaya*, *fbrsl1*, *grm4*, and *keap1a*), proteoglycan metabolism (*sulf2a*), small GTPase signaling (*plekhg4*), cell adhesion and migration (*nedd9*), cell growth and cycle regulation (*fhl3a*, *bcar1*, *fermt2*, *plekhg4*, and *phactr4a*), DNA reparation (*slx4ip*), and some zygotic genes (*tshz1* and *net1*) (Table 1). Heatmap analysis further showed that several representative genes, including bcor, tshz1, slx4ip, and mgat3a, displayed progressive DNA hypomethylation accompanied by increased transcriptional activity from PF-i to PF-iv (Figure 1D,E).

### 3.2. Candidate-Gene Expression Across PF Subtypes

**Figure 2:**
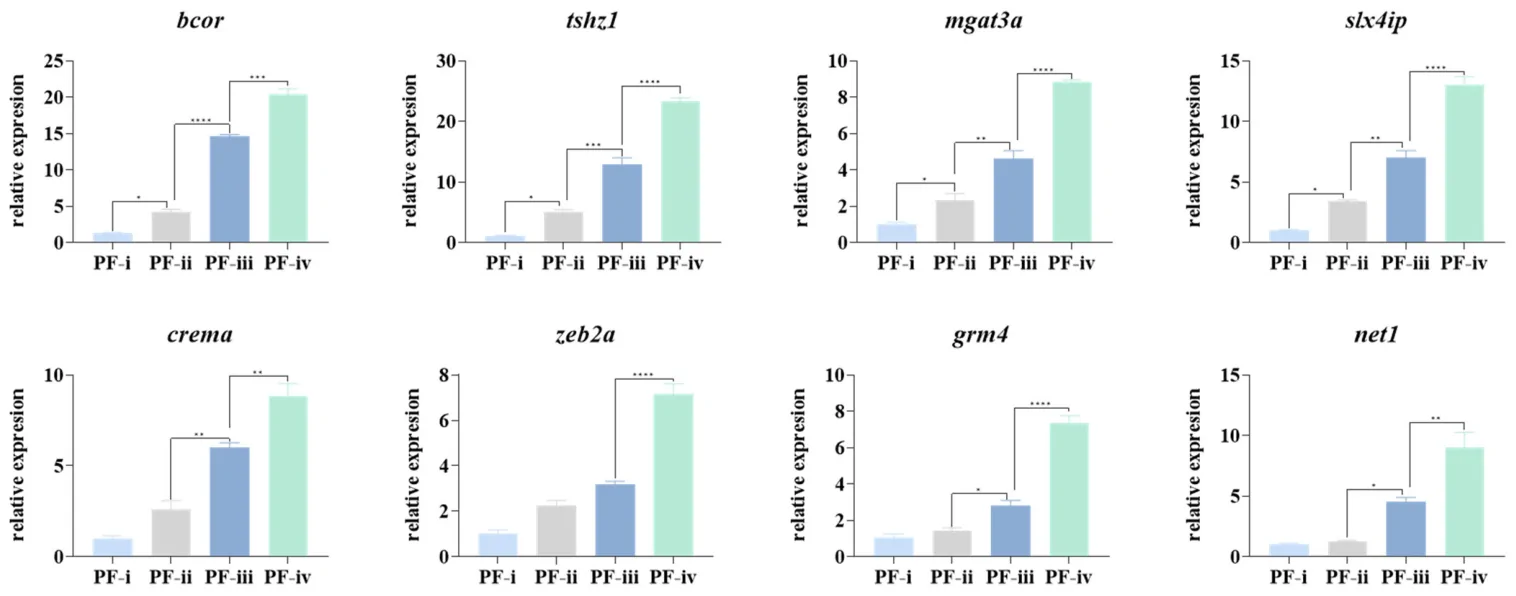
qRT-PCR validation of candidate driver genes during the development of four zebrafish PF subtypes. “*”: *p* < 0.05; “**”: *p* < 0.01; “***”: *p* < 0.001; “****”: *p* < 0.0001 (mean ± SD of relative expression; *n* = 3 for each group).

To validate the transcriptional profiles identified by RNA-seq analysis, the expression patterns of eight representative candidate genes were examined by quantitative real-time PCR (qRT-PCR) across four sequential PF subtypes (PF-i to PF-iv) (Figure 2). The expression of β-actin did not differ significantly across the PF-i to PF-iv stages, supporting its use as the reference gene (Figure S2). Consistent with the transcriptomic data, all selected genes exhibited a progressive increase in mRNA abundance during primary follicle development. Gene expression levels showed a slight increase at the PF-ii stage, followed by a marked elevation during the transition from PF-ii to PF-iii, and reached the highest levels at the PF-iv stage. Statistical analysis confirmed significant differences among developmental stages for most genes. These results supported the RNA-seq findings and further supported the association between DNA methylation remodeling and transcriptional activation during primary follicle maturation.

### 3.3. MAPK-Related Gene Expression and Protein Localization During PF Development

**Figure 3:**
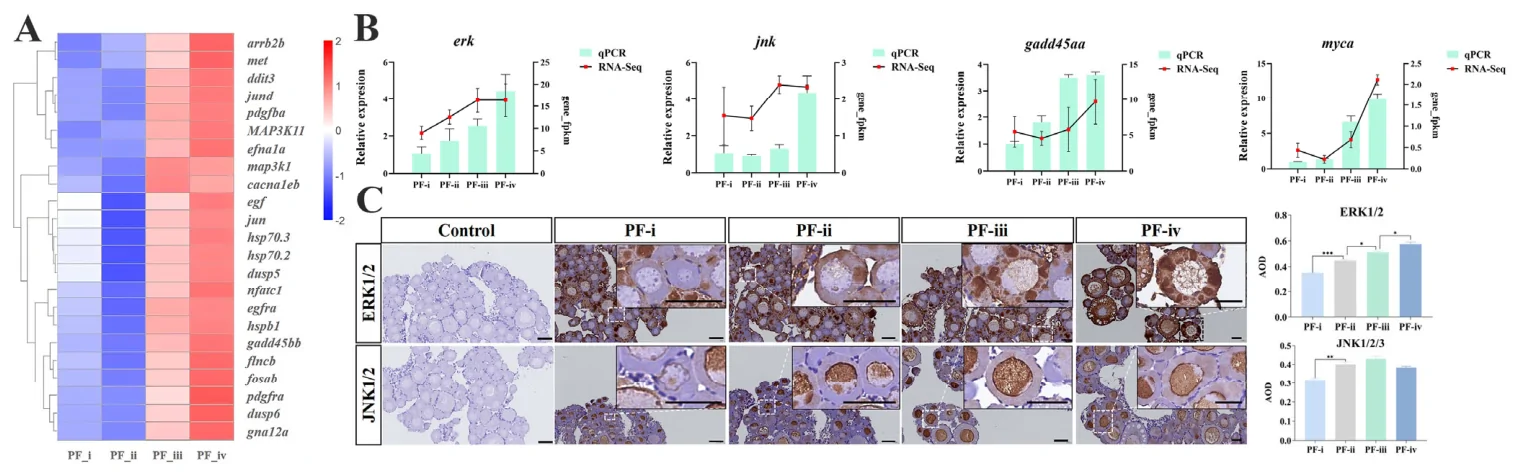
MAPK-related gene expression and total ERK/JNK localization associated with zebrafish PF development. (**A**) Heatmap of MAPK-related transcript expression. (**B**) qRT-PCR validation of the expression patterns of *erk*, *jnk*, *gadd45aa*, and *myca* identified from RNA-seq analysis. RNA-seq expression levels are shown as red lines (FPKM values), and qRT-PCR results are presented as green bars (relative mRNA expression levels). (**C**) ERK1/2 and JNK1/2 IHC images and the associated average optical density (AOD) plots shown to their right. Scale bar, 50 μm. “*”: *p <* 0.05; “**”: *p* < 0.01; “***”: *p* < 0.001; (mean ± SD of relative expression; *n* = 3 for each group).

Heatmap profiling of MAPK cascade-related transcripts further illustrated the dynamic transcriptional changes of core pathway genes across four primary follicle subtypes (Figure 3A). RNA-seq and qRT-PCR analyses showed increased expression of *erk*, *jnk*, *gadd45aa*, and *myca* during the PF-ii-to-PF-iii transition (Figure 3B). Immunohistochemical (IHC) images showed ERK staining predominantly in the oocyte cytoplasm and JNK staining mainly in oocyte nuclei. The associated optical-density plots are included within Figure 3C. Quantitative analysis of IHC signals using ImageJ software confirmed that both ERK and JNK protein levels significantly increased from PF-ii to PF-iii (Figure 3C).

### 3.4. Ovarian Morphology and Molecular Responses Following SCH772984 Exposure

**Figure 4:**
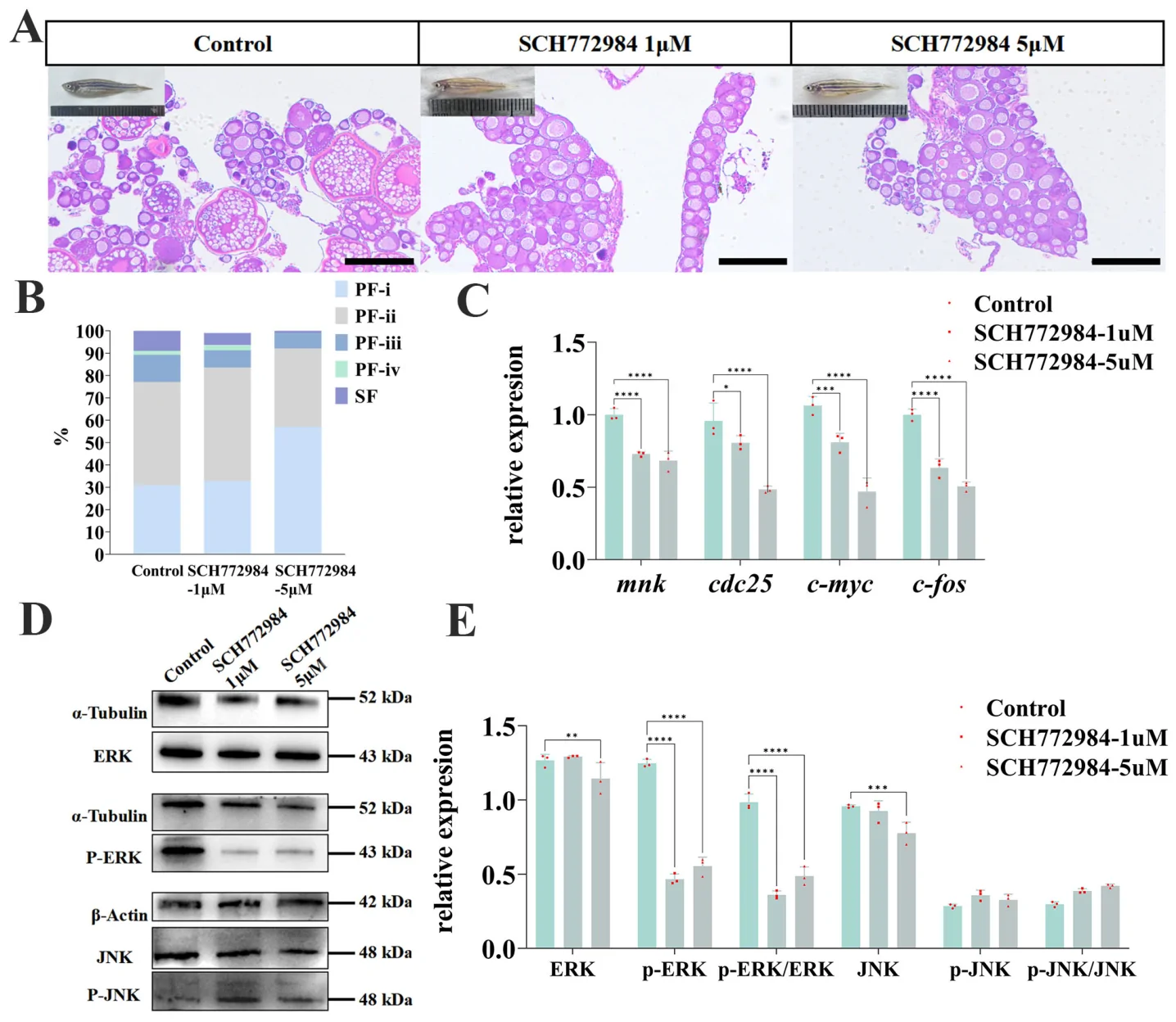
Ovarian morphology and molecular responses following SCH772984 exposure from 25 to 60 dpf. (**A**) Histological analysis of zebrafish ovaries following SCH772984 treatment. Scale bar, 100 μm. (**B**) Mean proportions of primary follicle subtypes (PF-i–PF-iv) and secondary follicles (SFs) in the vehicle-control and SCH772984-treated groups. These data are descriptive; no inferential statistical analysis was performed. (**C**) The *mnk*, *cdc25*, *c-myc*, and *c-fos* mRNA levels expression in ovary tissues of SCH772984-treated groups. (**D**,**E**) The ERK, p-ERK, JNK, and, p-JNK protein levels expression in ovary tissues of SCH772984-treated groups. In panels (**C**,**E**), data are presented as mean ± SD, with individual data points shown. Each point represents a pooled ovarian sample from three fish from one tank (*n* = 3 independent tanks per group). Each outcome was analyzed using one-way ANOVA followed by Bonferroni-adjusted comparisons of each inhibitor group with the vehicle control. Brackets indicate the comparisons shown. Asterisks denote adjusted *p* values: “*”: *p* < 0.05; “**”: *p* < 0.01; “***”: *p* < 0.001; “****”: *p* < 0.0001.

Zebrafish were exposed to SCH772984 from 25 to 60 dpf. Somatic growth parameters including body length and body mass were measured at 60 dpf to assess potential systemic effects of SCH772984 treatment. No significant differences in body length or body mass were observed across the DMSO control, 1 μM and 5 μM SCH772984 groups (one-way ANOVA, *p* > 0.05; Table 2). Ovaries from the vehicle-control group contained all four PF subtypes and SFs (Figure 4A). Histological observation revealed intact follicle progression in the vehicle control group, with all four subtypes of primary follicles (PF-i to PF-iv) readily detectable alongside abundant vitellogenic secondary follicles (SFs). In contrast, SCH772984 treatment increased the proportion of early-stage PF-i follicles, indicating a shift toward earlier follicle developmental stages (Figure 4A,B). These observations are consistent with impaired follicular progression during ERK inhibitor exposure.

qRT-PCR analysis showed that mRNA transcripts of four canonical ERK downstream targets (*mnk*, *cdc25*, *c-myc*, *c-fos*) were significantly downregulated in both SCH772984 treatment groups in a concentration-dependent manner (Figure 4C). Western blot analysis showed that total ERK and JNK abundance was significantly reduced in the 5 μM SCH772984 group compared with the vehicle control, whereas no significant differences were detected at 1 μM, while the level of phosphorylated ERK (p-ERK) was markedly reduced upon SCH772984 treatment, leading to a significantly decreased p-ERK/ERK ratio. By contrast, total p-JNK protein expression showed no obvious alteration (Figure 4D,E). These histological and molecular findings are consistent with disrupted primary follicle development under ERK inhibitor treatment.

## 4. Discussion

### 4.1. Candidate Regulators Associated with Early Primary Follicle Development

Primary follicle (PF) development is a complex developmental process involving coordinated epigenetic remodeling and transcriptional regulation [22,23,24]. Our previous study identified substantial molecular differences during the PF-ii-to-PF-iii transition [7]. By integrating those published transcriptomic and DNA methylation datasets, the present study identified genes with concurrent changes in expression and associated methylation. These candidates provide a basis for investigating the regulation of early follicular development, although the observed associations do not establish that methylation changes directly alter gene expression.

Among these candidates, transcriptional regulators, including *bcor*, *zeb2a*, *nfe2l1a*, *tfap2e*, *hivep2a*, and *crema*, may represent important upstream modulators of follicular cell fate determination. For example, *Bcor* functions as an epigenetic regulator involved in developmental gene expression, whereas *Zeb2* plays essential roles in cell differentiation and tissue remodeling [25,26,27]. The coordinated expression of these transcription factors suggests that extensive transcriptional reprogramming accompanies the PF-ii-to-PF-iii transition. Such reprogramming may help to coordinate the differentiation of follicular somatic cells, establish stable oocyte–somatic cell communication, and support the structural remodeling necessary for early follicle growth. Therefore, these transcription factors may act upstream of multiple signaling pathways to orchestrate the acquisition of developmental competence during primary follicle differentiation.

Protein modification-related genes, particularly *mgat3a*, may provide a potential link between protein glycosylation and intracellular signaling during follicle development [28]. MGAT3-mediated glycosylation has been reported to regulate ERK/MAPK signaling activity, suggesting that post-translational modification may influence follicular development by modulating key signaling pathways [29,30]. In addition, *slx4ip* was identified as a candidate in our integrative analysis; its function in zebrafish PF development remains to be tested [31,32]. Consistent with these findings, genes involved in cell adhesion and cytoskeletal organization (*bcar1*, *fermt2*, and *fhl3a*) were also differentially expressed, implying that coordinated regulation of genome stability, cell-cycle progression, and cellular remodeling may be required to support follicular differentiation and the acquisition of developmental competence [33,34,35]. The consistent expression of β-actin across PF subtypes supports its use for normalization (Figure S2), although validation with additional reference genes would further strengthen the qRT-PCR findings.

Collectively, these findings suggest that the PF-ii-to-PF-iii transition involves coordinated changes in epigenetic regulation, gene expression, and signaling-related processes. The integrative analysis identifies candidate regulators for further investigation of early follicular development in teleosts.

### 4.2. MAPK Signaling Pathway Regulates PF Development

The MAPK signaling pathway is widely recognized as a key regulator of folliculogenesis in vertebrates [36]. In mammals, MAPK/ERK signaling mediates the actions of gonadotropins and growth factors to regulate granulosa cell proliferation, differentiation, and follicular maturation [37,38]. In teleosts, MAPK signaling has likewise been implicated in mediating endocrine and local growth factor signaling to regulate follicular cell proliferation, oocyte maturation, and follicle development [3,39,40]. The present study extends these observations to early PF stages by combining subtype-associated molecular profiles with ovarian responses to ERK inhibitor exposure.

MAPK-related gene expression varied across PF subtypes, particularly during the PF-ii-to-PF-iii transition. Immunohistochemistry also revealed distinct distributions of total ERK and JNK within developing follicles. These findings provide spatial and developmental context for MAPK involvement in early follicular growth. However, because the antibodies detected total proteins, the staining patterns indicate protein distribution rather than pathway activation; stage-specific phosphoprotein localization would further resolve the activity of these pathways.

Genetic disruption of ERK1/2 signaling has been shown to impair follicular development and cause female infertility, underscoring the critical role of MAPK signaling in ovarian function [29,41]. In our study, SCH772984 exposure was associated with reduced ovarian ERK phosphorylation and lower mean proportions of advanced follicles. Together with the expression patterns across PF subtypes, these observations support a contribution of ERK-associated signaling to early follicular progression and complement previous work on terminal oocyte maturation in teleosts.

Interpretation of the pharmacological findings is limited by the use of chronic whole-animal exposure. Body length and body mass were recorded, but ovarian development was not compared among fish matched for size and developmental stage. Survival, feeding performance, gross abnormalities, and ERK responses in non-ovarian tissues were not assessed. The hepatic responses to ethinylestradiol reported in juvenile common carp [42] illustrate the relevance of extra-ovarian measurements in fish exposure studies, although they do not identify the mechanism of SCH772984 action. Thus, reduced ovarian ERK phosphorylation supports target engagement but does not establish an ovary-specific effect or exclude indirect and off-target contributions. Ovary-targeted genetic perturbation or rescue experiments would help determine whether ERK signaling directly regulates early follicular progression. 

### 4.3. Potential Interactions Among MAPK, Notch, and mTOR Signaling

Increasing evidence indicates that signaling pathways involved in folliculogenesis rarely function independently but instead form interconnected regulatory networks [43,44,45]. Previous studies in mammals have demonstrated that MAPK signaling regulates granulosa cell proliferation and differentiation partly through modulation of Notch pathway activity [46]. Similarly, in teleosts, MAPK signaling responds to endocrine and growth factor stimulation to regulate follicular development [47,48]. Our published zebrafish study implicated Notch and mTOR signaling in PF development, while the present findings support an association between ERK signaling and early follicular progression.

These observations provide a rationale for investigating possible interactions among MAPK, Notch, and mTOR during PF transitions. However, Notch and mTOR activity was not measured following SCH772984 exposure, and no pathway-interaction experiments were performed. Crosstalk among these pathways therefore remains a hypothesis. Combined perturbation and rescue experiments would be required to determine whether they act in parallel or through a shared regulatory mechanism.

## 5. Conclusions

Integrative reanalysis of published transcriptomic and DNA methylation datasets identified candidate genes associated with transitions among zebrafish primary follicle subtypes. MAPK-related expression and total ERK/JNK localization varied across these stages, while SCH772984 exposure was associated with reduced ovarian ERK phosphorylation and lower mean proportions of advanced follicles. The present effects were observed with a single inhibitor under nominal exposure. Together, these findings support the involvement of ERK-associated signaling in early follicular development and identify candidates for further functional investigation.

## Figures and Tables

**Table 1:** Candidate genes identified by integrated transcriptomic and DNA methylation analysis during zebrafish PF development.

Function	Comparative Group	Gene-Name	Methylation-Down/RNA-Up
Transcriptional regulation	PF-ii vs. PF-i	*bcor*	CG-down/RNA-up
	PF-iii vs. PF-ii	*hivep2a*	CG-down/RNA-up
		*tfap2e*	CG-down/RNA-up
		*Crema*	CG-down/RNA-up
		*zeb2a*	CG-down/RNA-up
		*nfe2l1a*	CG-down/RNA-up
Protein modification	PF-ii vs. PF-i	*btr07*	CG-down/RNA-up
		*mgat3a*	CHH-down/RNA-up
		*Atcaya*	CHH-down/RNA-up
	PF-iii vs. PF-ii	*fbrsl1*	CHH-down/RNA-up
	PF-iv vs. PF-iii	*grm4*	CHH-down/RNA-up
		*keap1a*	CHH-down/RNA-up
Proteoglycan metabolism	PF-ii vs. PF-i	*sulf2a*	CHH-down/RNA-up
Small GTPase signaling	PF-iii vs. PF-ii	*plekhg4*	CG-down/RNA-up
Cell adhesion and migration		*nedd9*	CHH-down/RNA-up
Cell growth and cycle regulation	PF-iii vs. PF-ii	*fhl3a*	CG-down/RNA-up
		*bcar1*	CG-down/RNA-up
		*fermt2*	CG-down/RNA-up
		*plekhg4*	CG-down/RNA-up
		*phactr4a*	CG-down/RNA-up
DNA reparation	PF-ii vs. PF-i	*slx4ip*	CG-down/RNA-up
Zygotic gene	PF-ii vs. PF-i	*tshz1*	CG-down/RNA-up
	PF-iii vs. PF-ii	*net1*	CG-down/RNA-up

**Table 2:** Body length and body mass of zebrafish at 60 dpf after exposure to SCH772984.

Group	Body Length (mm)	Body Mass (g)
Control	21.71 ± 0.31	0.22 ± 0.03
1 μM SCH772984	21.19 ± 0.62	0.21 ± 0.04
5 μM SCH772984	20.72 ± 0.51	0.20 ± 0.06

Note: Data are presented as mean ± SD. *n* = 3 independent tanks (biological replicates). Ten fish were sampled from each tank for body length and mass measurement. Fish were sampled at 60 dpf.

## Data Availability

The sequencing component reused published datasets (BioProject PRJNA1169999) [7]. All newly generated raw data, including qRT-PCR, Western blot, and histological/IHC data, are included within the article and Supplementary Materials of this manuscript.

## Supplementary Materials

The following supporting information can be downloaded at: https://www.mdpi.com/article/10.3390/ani16193078/s1, Figure S1. Observation of invitro follicles (previous row) and their histological sections (next row) fromPF-i, PF-ii, PF-iii, PF-iv, and SF. Scale bar, 20 μm. The follicles histological stained with hematoxylin and eosin. Figure S2. The relative expression levels of β-actin across the PF-i, PF-ii, PF-iii, and PF-iv stages. Table S1. Primer sequences used for polymerase chain reaction.
