# Supplementary material for: MAPK Signaling Contributes to Primary Follicle Development in Zebrafish (Danio rerio)
Source: Animals (Basel). 2026 Sep 30;16(19):3078. doi: 10.3390/ani16193078 (PMC13663377; doi:10.3390/ani16193078)
Supplement: Supplementary file 1 [file animals-16-03078-s001.zip › Supplementary Material (Figure).pdf]

Figure Legends

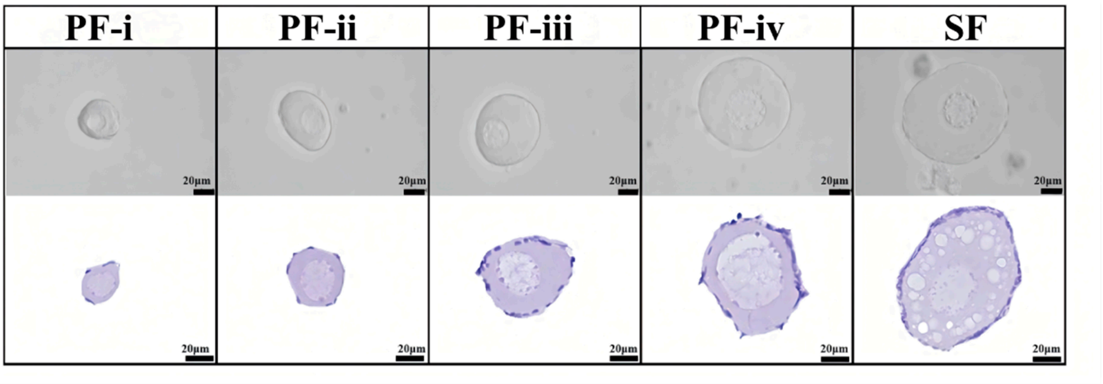

Figure S1. Observation of invitro follicles (previous row) and their histological sections (next row)fromPF-i, PF-ii, PF-iii, PF-iv, and SF.

Scale bar, 20 μm. The follicles histological stained with hematoxylin and eosin.

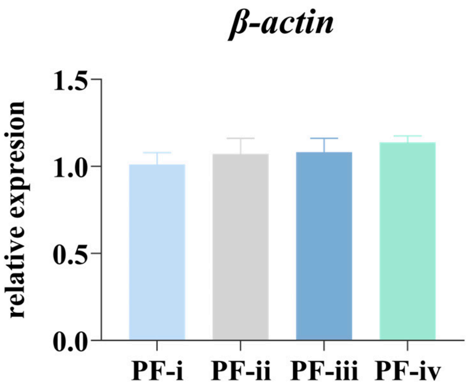

Figure S2. The relative expression levels of β-actin across the PF-i, PF-ii, PF-iii, and PF-iv stages.
