# Supplementary material for: MAPK Signaling Contributes to Primary Follicle Development in Zebrafish (Danio rerio)
Source: Animals (Basel). 2026 Sep 30;16(19):3078. doi: 10.3390/ani16193078 (PMC13663377; doi:10.3390/ani16193078)
Supplement: Supplementary file 1 [file animals-16-03078-s001.zip › Supplementary Material (Table).pdf]

Table S1. Primer sequences used for polymerase chain reaction.

Genes	Gene ID	Forward primers	Reverse primers	Amplicon lengths
bcor	402921	CCAAGATGACACAGATGGCAG	CCGTTGCTCTGAATCAGTTGGC	205bp
tshz1	369196	CAGATGCTTCCTGGTCGAGC	TGGCTGTTCGCGACGCTGTTG	118bp
mgat3a	553676	GGAGATCTCCTCTCCATCCAG	CTGATGGCTGGATTTCTGACG	118bp
slx4ip	393383	CTTGACACCACCGGTCCAAGA	GTTGGAATCGCTTGACACTGC	120bp
crema	555432	GCCACGCGCAAGAGAGAAGT	CTGGTTTCCAGCACGGCAAC	148bp
zeb2a	10003889	AGACTGCTGGGATCTGAGGGT	ATTTTCTTCGTCACCCCGGCA	108bp
grm4	567181	TTCAACTGCAAGCTCAGCCGA	CCTTTCCTTCCTGCTCGTAGG	109bp
net1	493607	GTACTAAGCGTGTGCGGCCT	CGTCACCCCGGAATGAGGC	120bp
erk	30707	GCCGGCAAACAACAGAAACCA	TCCATTCAGGTGGGCACTGGC	141bp
jnk	65236	CCGGTGACCACACCTACGAAT	CACTCAGGAGGGCACTGGCAA	134bp
gadd45aa	393548	GCTGCGAGAACGACATCAAC	GGGCACCCACTGATCCATACA	127bp
myca	30686	AGCCATCTTGAGGATCTTGGA	TCTTGGTCCGTTTCTGCTTCT	156bp
mnk	564117	AGGAGCCTGTACACCGTGTG	TCCAGGTTCACAGCCTTGAC	118bp
cdc25	57925	GGACCTGGAAGACATCGACA	GTGCTTGGTGTTGCTGATGG	132bp
c-myc	30686	ATGACCATGATTACGCCAAG	TCAGGTTCACGTATGCTGC	142bp
β-actin	57934	AGCCGAAAGAGAAATTGTCC	GAAACGCTCATTGCCGAT	122bp
